# Outcomes in Children With Umbilical Catheter-Related Portal Venous Thrombosis

**DOI:** 10.7759/cureus.78386

**Published:** 2025-02-02

**Authors:** Muharrem Cicek, Ozlem Kalaycik Sengul, Sumeyra Dogan, Seda Yilmaz Semerci, Gokhan Buyukkale

**Affiliations:** 1 Department of Pediatrics, Kanuni Sultan Suleyman Training and Research Hospital, Istanbul, TUR; 2 Department of Pediatric Gastroenterology, Kanuni Sultan Suleyman Training and Research Hospital, Istanbul, TUR; 3 Department of Pediatric Radiology, Kanuni Sultan Suleyman Training and Research Hospital, Istanbul, TUR; 4 Department of Neonatology, Kanuni Sultan Suleyman Training and Research Hospital, Istanbul, TUR

**Keywords:** children, newborn, portal hypertension, portal vein thrombosis, umbilical vein catheterization

## Abstract

Introduction: Umbilical vein catheterization (UVC) is commonly used in neonatal intensive care units (NICUs). However, it poses a significant risk for portal venous thrombosis (PVT). The aim of this study was to evaluate the patients with PVT due to UVC in a NICU in terms of the development of chronic thrombus, portal hypertension (PHT), and possible additional complications in the long-term outcome.

Methods: Demographic, clinical characteristics, laboratory findings, ultrasonography imaging, and treatment of patients aged three years and younger who were diagnosed with PVT after UVC in the NICU and followed up by the pediatric gastroenterology outpatient clinic of our hospital were evaluated retrospectively.

Results: A total of 29 pediatric patients were analyzed. Isolated left PVT was seen in 25 (86.2%) patients and two-sided/bilateral PVT in four (13.8%) patients during hospitalization in the NICU. In the follow-up of four patients with combined right and left PVT who received anticoagulant therapy, the thrombus disappeared completely in three patients, whereas it persisted in one patient with partial recanalized flow in the left portal vein.

Conclusion: Long-term follow-up of these patients is important for the management of PHT. Since UVC is commonly used in NICU, routine ultrasound evaluation of these patients for thrombosis can enable the diagnosis of PVT in the asymptomatic period.

## Introduction

Thrombosis is an abnormal clot plug formed by blood elements in the vein that prevents blood flow to the vein. It occurs when the balance between the procoagulant, fibrinolytic, and anticoagulant systems is disturbed. Contrary to what is known as a common clinical problem in adulthood, thrombosis can be seen in children, particularly in infants, and adolescents. The incidence of thrombosis has increased by 70% in children due to the survival of many critically ill patients who died early in the previous years, the increase in the frequency of use of catheters that trigger thrombosis, and the more widespread use of interventional procedures for diagnosis and treatment [1].

Despite the potential risk of thrombosis, umbilical vein catheter insertion remains a common practice in neonatal intensive care units (NICUs) as it ensures safe intravenous access, enables parenteral nutrition, facilitates fluid and medication administration, allows for blood sampling, and supports hemodynamic monitoring. Approximately 15% of newborns, primarily those weighing ≤1000 g at birth, undergo umbilical vein catheterization (UVC). Umbilical catheters can cause platelet aggregation and fibrin formation by damaging the vascular endothelium. Therefore, portal venous thrombosis (PVT) is a complication of UVC, and prehepatic portal hypertension (PHT) due to chronic thrombus can occur in the long term [2]. Although the incidence of umbilical catheter-related PVT is difficult to determine, it is reported to range from 1.3% to 43% for UVC-related PVT [3]. A multicenter study reported the incidence to be as high as 65%, attributed to the increase in the number of premature infants [4].

PVT in newborns is often asymptomatic, and spontaneous recanalization within an average of two months is the most characteristic feature. Neonatal PVT is the most important cause of PHT and PHT-related esophageal variceal bleeding in childhood [5]. Early diagnosis and treatment of PVT is crucial to avoid PHT and potentially serious long-term complications. Therefore, the aim of this study was to evaluate the patients with UVC-related PVT in terms of the development of chronic thrombus, PHT, and possible complications in the long-term follow-up.

This study was presented as an oral presentation at the 2^nd^ Eastern Pediatrics Congress (Doğu Pediatri Kongresi), which took place from September 29 to October 2, 2022, in Diyarbakir, Turkey.

## Materials and methods

This study retrospectively analyzed the demographic and clinical data of pediatric patients (≤3 years old) diagnosed with PVT in the NICU and followed up in our outpatient pediatric gastroenterology clinic from April 1, 2021, to March 31, 2022.

Patients with thrombosis secondary to catheterization were followed up without treatment in the case of a single-sided PVT, while anticoagulant therapy was initiated for main or two-sided PVT [6]. Each patient with PVT was followed up in the pediatric gastroenterology outpatient clinic due to the risk of PHT.

Study design

Between April 1, 2021, and March 31, 2022, the patients who were diagnosed with PVT following an umbilical vein catheter insertion during their NICU stay and were followed up by the pediatric gastroenterology outpatient clinic (≤3 years old) were included in this study. Infants with congenital and/or chromosomal abnormalities, newborns with an additional disease that may cause PVT, and patients with an additional disease-causing thrombophilia were excluded from the study.

Clinical information form

Demographic and clinical characteristics of patients, including gestational age, birth weight, mode of delivery, main reasons for hospitalization, hemogram, liver function tests, duration of NICU stay, data on umbilical vein catheter insertion and PVT diagnosis, ultrasound results and treatment, and if necessary, physical examination, were recorded.

The body weight and height measurements of the cases at the last follow-up (control) were evaluated and classified according to Neyzi et al.'s body weight and height reference values for age [7].

Patients were classified into two groups according to the catheter dwell time: <7 and ≥7 days.

Biochemical analysis

Hemogram (hemoglobin level, leukocyte count, and platelet count), serum total bilirubin, coagulation parameters (activated partial thromboplastin time (aPTT), prothrombin time (PT), and international normalized ratio (INR)), aminotransferases (aspartate transaminase (AST) and alanine transaminase (ALT)), and albumin values of all cases were noted during the pediatric gastroenterology outpatient clinic follow-ups. AST and ALT elevations <5 times the upper limit of normal were considered mild; 5-10 times the upper limit as moderate; and >10 times the upper limit as severe aminotransferase elevation. A platelet count below 150,000/μL was defined as thrombocytopenia, with values below 50,000/μL defined as severe thrombocytopenia. The lower limit of hemoglobin according to age and gender by the World Health Organization (WHO) was accepted as 11 g/dL for anemia in children aged 6-59 months [8].

Ultrasonographic imaging

Ultrasound evaluations of the cases in our study were performed by the same pediatric radiologist in both the NICU and pediatric gastroenterology outpatient clinic. The location of the thrombus, portal vein diameter, splenic vein diameter, portal vein flow velocity, liver size and parenchyma, spleen size, ascites, and collateral presence was recorded through abdominal and portal Doppler ultrasound imaging performed during hospitalization and at the time of admission to the pediatric gastroenterology outpatient clinic.

Statistical analysis

Statistical analysis of the study data was performed using the IBM SPSS Statistics program version 21.0 (IBM, Armonk, USA). Descriptive statistics were expressed as mean ± standard deviation (SD) or median (minimum-maximum) for discrete and continuous numerical variables, and number of cases and percentage (%) for categorical variables. Results were defined as statistically significant when p<0.05.

Ethics approval

This study was approved by the Ethical Committee of Kanuni Sultan Suleyman Training and Research Hospital, University of Health Sciences, Istanbul, Turkey (No. KAEK/2022.03.60).

## Results

A total of 29 pediatric cases, 18 (62.1%) male and 11 (37.9%) female, were included in this retrospective study (Table 1). The mean gestational age of the patients was 35 weeks (minimum 25 weeks, maximum 42 weeks), and their average birth weight was 2300 g (minimum 680 g, maximum 4430 g). About 18 (62.1%) patients were preterm infants. The birth weight of 18 patients was below 2500 g, 10 patients were between 2500-4000 g, and one patient was over 4000 g. Six of the 18 patients with a birth weight below 2500 g had very low birth weight (<1500 g). In the last pediatric gastroenterology outpatient follow-up of the patients, the mean age was found to be 19.3 months (range: 5-35 months, median: 22 months). The mean age of the male cases was 17.8 months, and the girls were 21.1 months old. When the reasons for admission to the NICU were evaluated, 18 (62.1%) patients had prematurity and accompanying respiratory distress (transient tachypnea of the newborn, congenital pneumonia, etc.), three (10.3%) had sepsis, two (6.8%) had hypoglycemia, one (3.5%) had complete exchange transfusion due to neonatal jaundice, three (10.3%) had perinatal asphyxia, one (3.5%) had dehydration, and one (3.5%) had a history of duodenal atresia. UVC was performed within the first two days in 26 patients (89.7%). The number of newborns with an umbilical vein catheter dwell time of less than seven days was two (6.9%), while the number of infants with an umbilical vein catheter dwell time of seven days or more was 27 (93.1%) (Table 1).

**Table 1:** Neonatal information of patients with PVT PVT: Portal venous thrombosis

Neonatal characteristic	Value	n (%)
Sex	Male	18 (62.1)
	Female	11 (37.9)
Birth weight (g)	<1500	6 (20.7)
	1500-2500	12 (41.4)
	2500-4000	10 (34.5)
	>4000	1 (3.4)
Gestational age (weeks)	<37	18 (62.1)
	≥37	11 (37.9)
Catheter insertion time (postnatal day of life)	<2	26 (89.7)
	≥2	3 (10.3)
Catheter dwell time (days)	<7	2 (6.9)
	≥7	27 (93.1)

In this study, although PVT development rates appeared higher in preterm infants, there was no statistically significant difference in thrombus status between initial and follow-up ultrasonographic findings in both preterm and term infants (p>0.05). Similarly, no significant association was found between UVC duration (≥7 days vs. <7 days) and PVT development rates based on initial ultrasonographic results (p=0.634). Additionally, PVT development rates did not significantly differ by gender (p=0.227) or birth weight groups (p=0.900). These findings suggest that factors such as prematurity, UVC duration, gender, and birth weight may not independently influence PVT development rates or thrombus resolution in this cohort.

During the NICU stay, AST levels were found to be normal in most cases (n=24, 86.2%), but a slight elevation was found in four cases. While ALT was at a normal level (93.1%) in most patients in the NICU, it was found to be slightly elevated in two patients during the NICU stay. Transaminase levels were found to be normal for all cases during the follow-up at the pediatric gastroenterology outpatient clinic. The mean AST value of the patients at the time of diagnosis was 64 U/L (minimum 27 U/L, maximum 164 U/L) and the mean AST value at the last outpatient follow-up was 37 U/L (minimum 16 U/L, maximum 50 U/L). The mean ALT value of the patients at the time of diagnosis was 15 U/L (minimum 3 U/L, maximum 80 U/L), and the mean ALT value at the last outpatient follow-up was 17 U/L (minimum 9 U/L, maximum 41 U/L). In this study, AST and ALT levels were compared between preterm and term infants at the time of PVT diagnosis and during follow-up (control). The results showed no statistically significant differences in initial AST levels (p=0.382) or control AST levels (p=0.183) between the groups. However, initial ALT levels were significantly higher in term infants compared to preterm infants (p=0.019). Although control ALT levels also tended to be higher in term infants, this difference did not reach statistical significance (p=0.090). These findings suggest that ALT levels at the time of PVT diagnosis may differ significantly between term and preterm infants, but this difference appears to diminish over time. In contrast, AST levels showed no significant differences between the groups.

Two patients had an elevated INR (6.9%) at the time of PVT diagnosis. INR values were within normal ranges in all patients during outpatient clinic follow-ups. All patients had normal albumin values at both diagnosis and follow-up. Bilirubin levels were also normal for all patients during outpatient follow-up. Only one patient with complete blood exchange due to neonatal jaundice at the time of diagnosis had mild anemia. At outpatient follow-up, five patients had mild anemia (lowest hemoglobin 9.6 mg/dL). At the time of PVT diagnosis, three patients had platelets below 150,000/mm³, which was thought to be due to sepsis and mild leukocytosis that was detected in these patients. At outpatient follow-up, platelet and leukocyte values were found to be within normal ranges in all patients. In this study, platelet levels of preterm and term infants were compared at the time of PVT diagnosis and during follow-up (control) measurements. No statistically significant difference was found in platelet levels between preterm and term infants at the time of PVT diagnosis or during follow-up (diagnosis: p=0.401; control: p=0.390). These findings suggest that platelet levels in preterm infants tend to be lower compared to term infants; however, these differences do not reach statistical significance. Further studies are required to assess the clinical implications of platelet levels in neonates.

In ultrasonographic evaluations, hepatomegaly, splenomegaly, ascites, collateral development, liver atrophy, PHT, and portal biliopathy were not observed in any patient, either at the time of diagnosis or during outpatient follow-up visits. In addition, liver parenchyma was homogeneous in all cases. None of the patients had PHT, esophageal varices, hypersplenism, hepatomegaly, or splenomegaly upon physical examination.

In the growth curve, there were two patients (6.9%) whose weight-for-age was below the 3^rd^ percentile and one subject (3.4%) whose weight-for-age was between the 3^rd^-10^th^ percentile, while there was one subject (3.4%) whose height-for-age was below the 3^rd^ percentile and two subjects (6.9%) whose height-for-age was between the 3^rd^-10^th^ percentile. All patients with malnutrition were noted to be preterm infants with a gestational age below 28 weeks.

In the ultrasound controls performed after UVC, left PVT was detected in 25 (86.2%) patients, while two-sided PVT was detected in four (13.8%) patients. A total of 25 patients (86.2%) did not receive anticoagulant or antithrombotic treatment for PVT, while all four patients with two-sided PVT received low molecular weight heparin (LMWH) (mean: two months, minimum-maximum: 1-3 months). None of the patients developed treatment-related complications.

When the patients with and without treatment were analyzed separately, it was found that the thrombus completely disappeared in 16 (55.2%) patients, partial thrombus persisted in the left portal vein branches in 10 (34.5%) patients, and partial recanalized flow in the left PVT was observed in three (10.3%) patients, followed up without treatment due to isolated left PVT (Table 2). In the follow-up of four patients with combined right and left PVT who received anticoagulant therapy, the thrombus disappeared completely in three patients, whereas it persisted in one patient with partial recanalized flow in the left portal vein. There was no statistically significant difference in the complete resolution or persistence of thrombus between patients who received anticoagulant therapy and those who did not (p=1.0).

**Table 2:** Doppler ultrasound evaluation results PVT: Portal venous thrombosis

PVT location	Neonatal Doppler ultrasound, n (%)	Follow-up Doppler ultrasound, n (%)
Left	25 (86.2)	13 (44.8)
Right	0 (0)	0 (0)
Left + right	4 (13.8)	0 (0)
No thrombosis	0 (0)	16 (55.2)

Tests for the causes of thrombophilia (Factor-V Leiden, prothrombin, methylenetetrahydrofolate reductase (MTHFR), plasminogen activator inhibitor-1 (PAI-1), and Factor-XIII mutations) were performed in four patients with two-sided PVT, and the results were reported as normal.

## Discussion

In this study, we evaluated patients with PVT thrombosis secondary to UVC during NICU admission and followed up by the pediatric gastroenterology outpatient clinic in the short and long term. The incidence of thrombosis is five times higher in neonates compared to the pediatric age group, and the use of arterial/venous catheters, which is the most important cause in the etiology, is thought to contribute to this increase [9]. Although PVT is not common in childhood, early diagnosis and treatment are crucial due to the associated morbidity and mortality. Assessment of PVT existence following UVC, which is widely used in neonatal units, through routine ultrasound examination is essential in detecting cases that can benefit from treatment [1,10]. Pediatric patients with splenomegaly and/or upper gastrointestinal (GI) bleeding findings should be questioned about their history of NICU hospitalization and catheter insertion; therefore, chronic PVT should be investigated as a potential etiology during the asymptomatic period. Early diagnosis and treatment can facilitate regular follow-up and management of these patients, avoiding potential serious and fatal complications.

In adults, any risk factor for thrombosis is not seen in 25-50% of all venous thrombi, whereas in children, at least one risk factor is found in more than 95% of cases with thrombi [11]. While multifactorial etiology plays a role in the development of PVT, the most common causes are umbilical artery/vein catheterization, neonatal sepsis, and omphalitis; however, the cause of thrombosis remains unidentified in approximately half of the children with thrombosis [12,13]. The risk of thrombosis varies depending on the diameter of the catheter, length of stay, location, and the content of the infusions administered through the catheter, with the risk of thrombosis increasing in direct proportion to the length of stay of the catheter [6]. In our study, in 27 (93.1%) newborns, the umbilical vein catheter dwell time was found to be seven days or more, and the duration was observed to increase the risk of thrombosis in accordance with the literature [5]. The mean gestational age of the patients was 35 weeks, and the majority (62.1%) were preterm infants. Given that prematurity itself is considered an individual risk factor for PVT, meticulous attention should be given to this group of patients with additional risk factors such as UVC [5]. 

Since the liver is supplied by the hepatic artery in addition to the portal vein, liver synthesis functions are usually not impaired in pediatric patients with PVT, and parameters such as AST, ALT, bilirubin, albumin, and coagulation factors are usually within normal ranges, in contrast to adult cases [14]. AST and ALT values were found to be normal in 86.2% and 93.1% of the patients at the time of diagnosis in the NICU, respectively. In two patients with perinatal asphyxia who had mild elevations in both AST and ALT, these levels returned to normal ranges after treatment. Therefore, elevated liver enzymes are thought to be associated with impaired liver perfusion secondary to asphyxia. During follow-up at the pediatric gastroenterology outpatient clinic, the liver enzymes of the patients were within the normal range and were consistent with the literature.

In patients with PVT, PHT and moderate hypersplenism along with leukopenia and thrombocytopenia can be detected in approximately 40-80%, with PHT observed during long-term follow-up [15]. In a study by Ferri et al., hypersplenism leading to thrombocytopenia was found in 61.8% of patients followed for PVT [16]. Thrombocytopenia, which was thought to be related to sepsis at the time of diagnosis, was detected in three of our patients during the NICU period, although thrombocytopenia and leukopenia were not observed during subsequent follow-up.

Since the umbilical vein catheter passes through the ductus venosus associated with the left portal vein, a considerable portion of catheter-related PVTs is observed in the left portal vein [6]. In this study, left PVT was identified in 25 (86.2%) patients, which aligns with existing literature. During follow-up, asymptomatic left liver lobe atrophy was reported in 28% of the patients with PVT in the neonatal period [17]. However, left lobe atrophy was not detected in any of our patients during follow-up.

Chronic PVT is associated with growth and developmental retardation in children, although the predisposing factors remain unclear [15]. Growth and development in these children can be suppressed due to chronic anemia, hepatotropic hormone deprivation resulting from decreased liver blood flow, deterioration in liver synthesis functions, and malabsorption [15,18]. Growth retardation (below the 5^th^ percentile for weight-for-age and height-for-age) was reported in approximately half of the patients with PVT during long-term follow-up [19]. In our study, weight-for-age was below the 3^rd^ percentile in two (6.9%) patients and height-for-age was below the 3^rd^ percentile in one (3.4%) patient. All of these patients with malnutrition were preterm infants with very low birth weight. Simultaneous evaluation of these patients in terms of nutrition and growth during their follow-up in the pediatric gastroenterology outpatient clinic, along with the regulation of their daily caloric intake by adding enteral nutrition solutions in necessary cases, was thought to be the reason for the low rates of growth and developmental retardation in our study. Based on the relevant literature, we suggest that it is important to monitor the height and weight of patients with PVT at each outpatient visit in order to support growth and development when necessary [15,18,19].

Spontaneous resolution of some PVT cases has been reported in the literature, with 60-70% of neonatal PVT cases showing spontaneous resolution in the neonatal period [6,20-22]. However, a 2011 systematic review covering all age groups indicated that approximately 80% of acute PVT cases do not improve without treatment [23]. Specific therapeutic management is mandatory in selected cases of PVT, although the safer treatment options and optimal duration of treatment are still unknown [24,25]. Due to the rarity of PVT in neonates, there are no controlled studies of therapeutic trials, and the optimal duration of anticoagulation treatment has not been established [6,26]. Since catheterization-associated PVT in the neonatal period is known to resolve spontaneously, the benefit of anticoagulation therapy remains unclear. In cases of thrombosis associated with UVC, the catheter should be removed if possible. Conservative treatment with supportive care and serial monitoring is recommended for patients with left PVT, whereas anticoagulant therapy is advised for patients with main or right PVT [6]. LMWH is preferred over other anticoagulant drugs due to its lower risk of major bleeding [5,6,27]. In a study conducted in neonates, clinically silent PVT was detected by ultrasound in 17 (34%) out of 50 neonates, and complete or partial resolution of the thrombus was observed in 13 (76.5%) of the 17 infants with UVC-associated PVT who did not receive any treatment [28]. In another study, thrombus was completely resolved in half of the 36 newborns with PVT who were followed up without anticoagulant treatment [29]. In our study, 25 patients (86.2%) did not receive anticoagulant or antithrombotic therapy for left PVT, whereas all four patients with combined left and right PVT received LMWH therapy, with a mean duration of treatment of two months. Complete resolution of the thrombus was detected in 52% of patients with left PVT who were followed up without treatment and in 75% of patients who received LMWH treatment for both right and left PVT. In one patient who received treatment, partial recanalized flow was observed and partial recovery was detected. However, no treatment-related complications were seen during this study. PHT is the most serious long-term complication associated with PVT. One study found that PHT was diagnosed an average of 5.7 years following the acute event [30]. Research and case reports in the literature underscore the importance of long-term follow-up for infants with PVT [3,25].

Study limitations

In our study, the patients' intensive care requirements, birth weight, gestational age, and duration of catheter stay were similar to those reported in the literature. However, no comparison could be made in terms of other risk factors associated with UVC (catheter diameter, location, and infusions administered) due to the retrospective design of the study. Therefore, the major limitation of our research pertains to its retrospective nature. Due to the disruption of control examinations during the COVID-19 pandemic, our study was extended to a maximum duration of three years. Since thrombosis persists, even partially, in approximately half of the patients with left PVT, we recommend extending follow-up periods in accordance with the literature. 

## Conclusions

Since UVC is commonly used in NICUs, routine ultrasound evaluation for patients at high risk of thrombosis can facilitate the diagnosis of PVT during the asymptomatic period. Anticoagulant treatment for patients with bilateral PVT is significant in terms of long-term complications related to PHT. In cases of left PVT, thrombosis can persist, albeit partially, even if most of the thrombosis resolves spontaneously without treatment. There are a limited number of studies on UVC-related PVT during the neonatal period. Therefore, controlled randomized studies with larger series and longer follow-up periods are needed to make clear recommendations on the subject.
